# Bisphenol A in Thermal Paper Receipts: An Opportunity for Evidence-Based Prevention

**DOI:** 10.1289/ehp.1104004

**Published:** 2012-01-01

**Authors:** Andrea W. Schwartz, Philip J. Landrigan

**Affiliations:** Department of Preventive Medicine, Mount Sinai School of Medicine, New York, New York, E-mail: phil.landrigan@mssm.edu

The recent report by Taylor et al. (2011) on the pharmacokinetics of bisphenol A (BPA) emphasizes the similarities between humans, monkeys, and mice in the metabolism of this ubiquitous and potentially toxic synthetic chemical. The authors suggested that human exposure to BPA may be “much higher than previously assumed.” They observed that a potentially important nonfood source of exposure to BPA may be the thermal paper used in cash register receipts.

BPA is found in receipt paper (Mendum et al. 2010) and appears to transfer readily from receipts to skin (Biedermann et al. 2010) and to be absorbed transdermally (Zalko et al. 2011). Retail workers, who likely have more frequent exposure to cash receipts containing BPA than other Americans, have been found to have elevated levels of urinary BPA (Lunder et al. 2010). BPA has been shown to be capable of crossing the placenta (Balakrishnan et al. 2010) and to be toxic during early mammalian development (vom Saal and Hughes 2005). This toxicity is relevant to humans, given the similarities in BPA metabolism observed across species by Taylor et al. (2011). Prenatal exposure of human infants to BPA has been associated with behavioral anomalies (Braun et al. 2009).

There is a sense of déjà vu about this story: In the 1970s polychlorinated biphenyls (PCBs) were widely used in carbonless copy paper (Erickson and Kaley 2011). PCBs were shown to be absorbed through the skin (Carpenter 2006), and prenatal exposures to PCBs were subsequently shown to cause irreversible brain injury to developing fetuses, which resulted in permanent loss of IQ (intelligence quotient) and alterations in behavior (Jacobson and Jacobson 1997). This exposure ended when the manufacture of PCBs was banned in the United States in 1976.

The research of Taylor et al. (2011) contributes to our understanding of the potential harms to the developing fetus from BPA. These findings underscore the need to develop a new U.S. chemical policy that would require toxicological testing of widely used chemicals already on the market and premarket safety testing of all proposed new chemicals (Landrigan and Goldman 2011). The time to presume that chemicals are safe until they are proven beyond all doubt to cause injury to America’s children is past. While research into the effects of exposure to BPA continues, we have an opportunity to act today on the basis of the available evidence to remove BPA from thermal paper, as we strive to protect the health and future intelligence of America’s children.
